# Supplementary material for: Choosing Wavelet Methods, Filters, and Lengths for Functional Brain Network Construction
Source: PLoS One. 2016 Jun 29;11(6):e0157243. doi: 10.1371/journal.pone.0157243 (PMC4927172; doi:10.1371/journal.pone.0157243)
Supplement: S1 File — (PDF) [file pone.0157243.s001.pdf]

Supplementary Material for "Choosing Wavelet Methods, Filters, and Lengths for Functional Brain Network Construction"

Zitong Zhang[1,2], Qawi K. Telesford[2], Chad Giusti[2,3], Kelvin O. Lim[4], Danielle S. Bassett[2,5*]

1 Department of Biomedical Engineering, Tsinghua University, Beijing 100084, China
2 Department of Bioengineering, University of Pennsylvania, Philadelphia, PA 19104, USA
3 Warren Center for Network and Data Sciences, University of Pennsylvania, PA 19104, USA
4 Department of Psychiatry, University of Minnesota, Minneapolis, MN 55455, USA
5 Department of Electrical and Systems Engineering, University of Pennsylvania, Philadelphia, PA 19104, USA

* dsb@seas.upenn.edu

Supplemental Methods

Wavelet Coefficients and Wavelet Details

The jth level MODWT wavelet and scaling coefficients for signal $\mathbf{X}$ with length N are defined, respectively, as:

$$\tilde{W}_{j,t} = \Sigma_{l=0}^{L_j-1} \tilde{h}_{j,l} X_{t-l \bmod N} \text{ and } \tilde{Y}_{j,t} = \Sigma_{l=0}^{L_j-1} \tilde{g}_{j,l} X_{t-l \bmod N},$$

where $\{\tilde{h}_{j,l}\}$ and $\{\tilde{g}_{j,l}\}$ are the jth level MODWT wavelet and scaling filters, and L_j is their common length. In matrix form, the above expression can also be written as:

$$\tilde{\mathbf{W}}_j = \tilde{W}_j \mathbf{X} \text{ and } \tilde{\mathbf{Y}}_j = \tilde{Y}_j \mathbf{X}.$$

Together, the wavelet and scaling coefficients form an energy decomposition and ANOVA of the original signal:

$$||\mathbf{X}||^2 = \Sigma_{j=1}^{J_0} ||\tilde{\mathbf{W}}_j||^2 + ||\tilde{\mathbf{Y}}_{J_0}||^2.$$

On the other hand, the jth level details and smooths are defined by:

$$\tilde{D}_j = \tilde{W}_j^T \tilde{\mathbf{W}}_j \text{ and } \tilde{S}_j = \tilde{Y}_j^T \tilde{\mathbf{Y}}_j.$$

Together, they define a multiresolution analysis (MRA) of the signal:

$$\mathbf{X} = \Sigma_{j=1}^{J_0} \tilde{D}_j + \tilde{S}_{J_0}.$$

Wavelet Coherence

The wavelet coherence of two signals x and y is defined as [**?**]:

$$G[Cx^*(a,b)Cy(a,b)]/[\sqrt{G(|Cx(a,b)|^2)}\sqrt{G(|Cy(a,b)|^2)}],$$

where $Cx(a,b)$ and $Cy(a,b)$ represent the continuous wavelet transform (CWT) of x and y, respectively, and G stands for a smoothing operator in time and scale. In this study, we have averaged 10 adjacent time points and have not smoothed in scale. Note that scale 1∼4 correspond to $a = 1$, 2, 4, and 8, respectively. To calculate the value of connectivity between two brain regions, we then compute the average of this time course.

Supplemental Results

Network Diagnostic Values Across Frequency Bands

In the main manuscript, we report network diagnostic values obtained from functional brain networks constructed from scale 2 wavelet coefficients. In particular, we report that wavelet filter type (D, LA, and C) has little effect on estimated diagnostics for identical wavelet lengths. Here we show similar results for functional brain networks constructed from scale 1 (approximately 0.125–0.25 Hz; see Fig. 1), scale 3 (approximately 0.03–0.06 Hz; see Fig. 2), and scale 4 (approximately 0.015–0.03 Hz; see Fig. 3) wavelet coefficients.

Network Diagnostic Values Across Densities

In the main manuscript, we report network diagnostic values obtained from functional brain networks constructed from the 30% strongest connections. Here we show similar results for functional brain networks constructed from the 25% strongest connections (see Fig. 4) and the 35% strongest connections (see Fig. 5). These results are both quantitatively and qualitatively similar to those reported for networks constructed from the 30% strongest connections, suggesting that our conclusion regarding these data (namely that wavelet filter type – D, LA, and C – has little effect on estimated diagnostics for identical wavelet lengths) is robust to small variations in network density.

Frequency Dependence of Wavelet Filter Length Effects

In the main manuscript, we observe that the length of the wavelet filter differentially effects network diagnostics obtained from functional brain networks constructed from scale 2 wavelet coefficients; some diagnostics are affected significantly (such as the modularity index), and other diagnostics are affected very little (such as the characteristic path length). We quantify this differential sensitivity using a set of repeated measures ANOVA (ANalysis Of VAriance), for each diagnostic and each type of wavelet filter, where wavelet length was treated as a categorical factor, and diagnostic type was treated as a repeated measure.

Here we ask whether this differential sensitivity is dependent on frequency by performing the same set of ANOVAs for functional brain networks constructed from scale 1 (Tab. 1), scale 3 (Tab. 2), and scale 4 (Tab. 3) wavelet coefficients. The pattern of results in the functional brain networks constructed from scale 1 wavelet coefficients is very consistent with that observed for functional brain networks constructed from scale 2 wavelet coefficients (compare Tab. 1 in this supplement with Tab. 2 in the main manuscript). In both cases, the mean correlation coefficient, variance of the correlation coefficients, and number of communities tend to be sensitive to wavelet length across all wavelet filter types (D, LA, and C). Observed effects in global efficiency, local efficiency, and modularity are less consistent across the two frequency bands.

Interestingly, in functional brain networks constructed from scale 3 wavelet coefficients, we observe that only one network diagnostic shows a significant wavelet length effect, and that is the variance of the correlation coefficients. In functional brain networks constructed from scale 4 wavelet coefficients, we observe robust main effects of wavelet length on the mean correlation coefficient, the variance of the correlation coefficients, and the modularity index.

In summary, the variance of the correlation coefficients is affected by wavelet length across all three wavelet types (D, LA, and C), and across all frequency bands examined (associated with scale 1, 2, 3, and 4 wavelet coefficients). Other network diagnostics showed differential sensitivity to wavelet length in the 4 frequency bands.

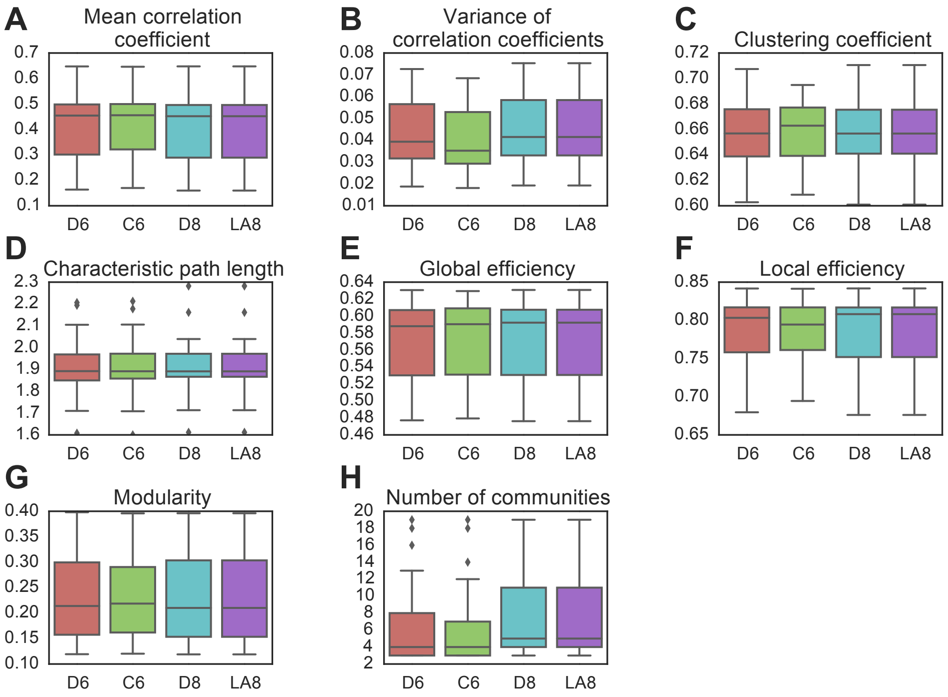

Fig 1. Effect of Wavelet Filter on Network Diagnostics in wavelet scale 1 between pairs of wavelet filters with the same length. *(A, B)* Weighted network diagnostics including *(A)* mean correlation coefficient and *(B)* variance of correlation coefficients. *(C–F)* Binary network diagnostics calculated at a graph density of 30% obtained through a cumulative thresholding procedure, including *(C)* the clustering coefficient, *(D)* characteristic path length, *(E)* global efficiency, *(F)* local efficiency, *(G)* modularity index Q, and *(H)* the number of communities. Boxplots indicate the median and quartiles of the data acquired from 29 health subjects.

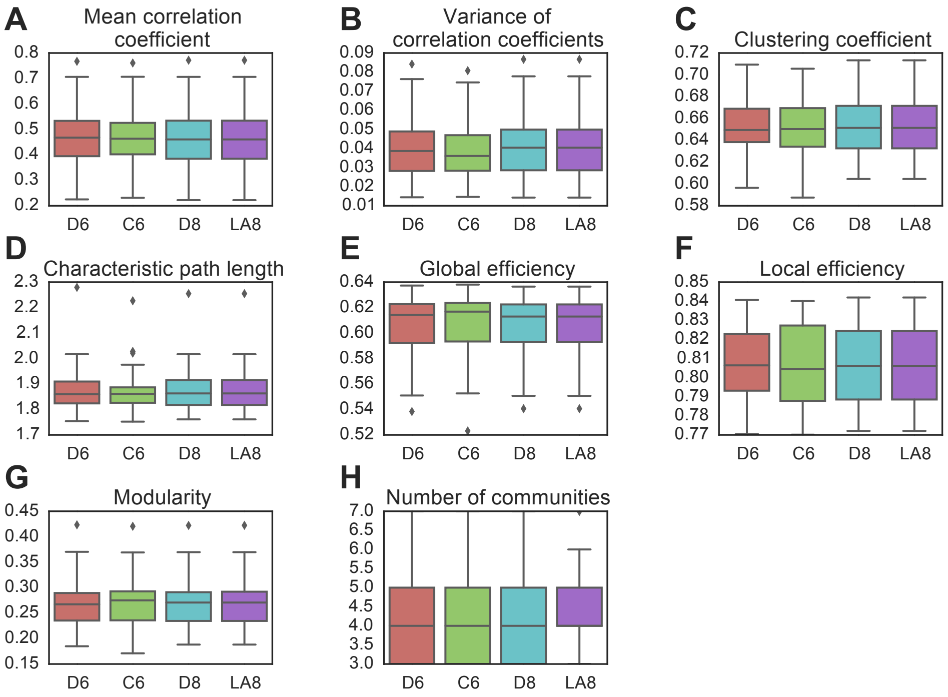

Fig 2. Effect of Wavelet Filter on Network Diagnostics in wavelet scale 3 between pairs of wavelet filters with the same length. *(A, B)* Weighted network diagnostics including *(A)* mean correlation coefficient and *(B)* variance of correlation coefficients. *(C–F)* Binary network diagnostics calculated at a graph density of 30% obtained through a cumulative thresholding procedure, including *(C)* the clustering coefficient, *(D)* characteristic path length, *(E)* global efficiency, *(F)* local efficiency, *(G)* modularity index Q, and *(H)* the number of communities. Boxplots indicate the median and quartiles of the data acquired from 29 health subjects.

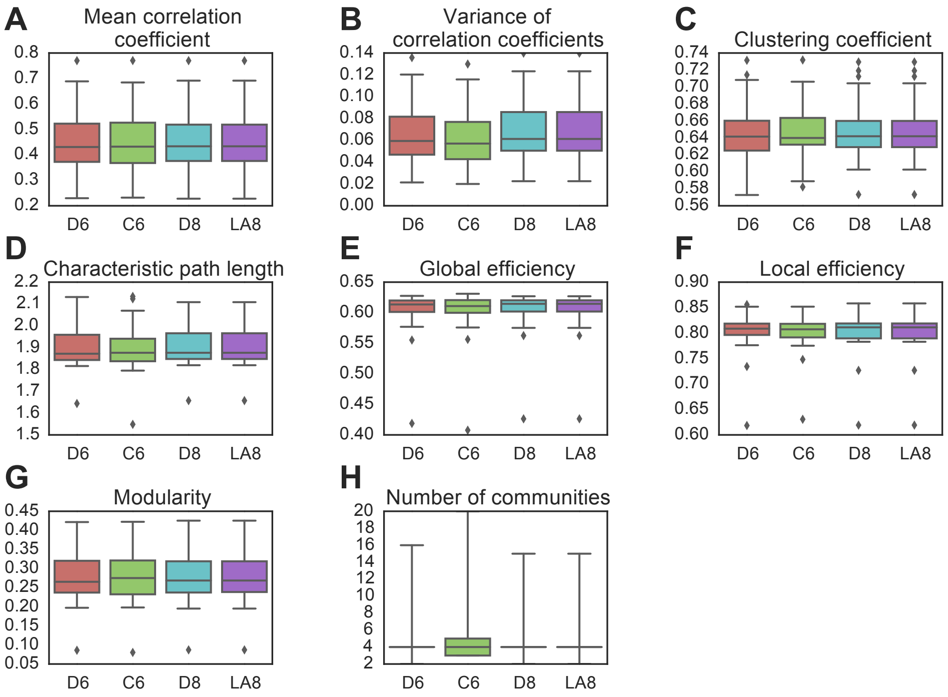

Fig 3. Effect of Wavelet Filter on Network Diagnostics in wavelet scale 4 between pairs of wavelet filters with the same length. *(A, B)* Weighted network diagnostics including *(A)* mean correlation coefficient and *(B)* variance of correlation coefficients. *(C–F)* Binary network diagnostics calculated at a graph density of 30% obtained through a cumulative thresholding procedure, including *(C)* the clustering coefficient, *(D)* characteristic path length, *(E)* global efficiency, *(F)* local efficiency, *(G)* modularity index Q, and *(H)* the number of communities. Boxplots indicate the median and quartiles of the data acquired from 29 health subjects.

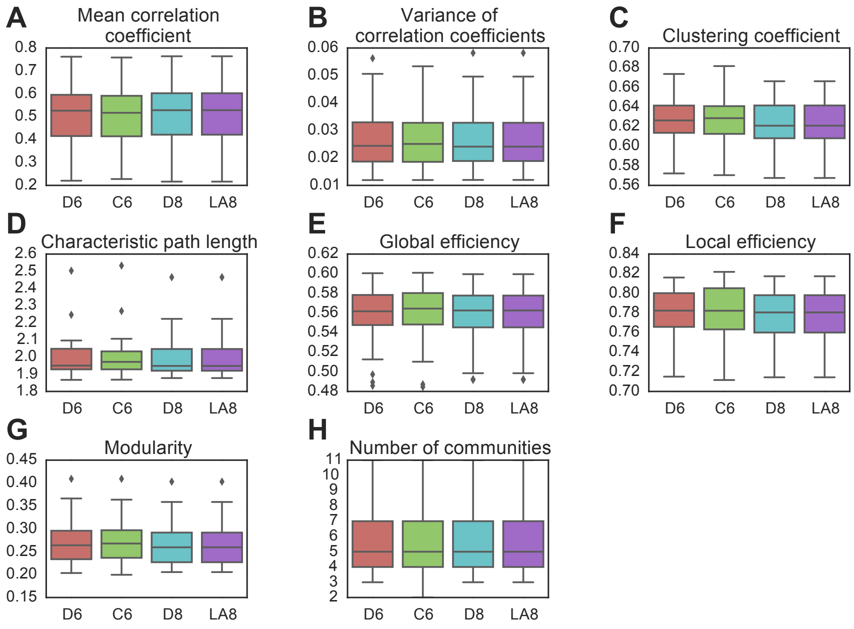

Fig 4. Effect of Wavelet Filter on Network Diagnostics in wavelet scale 2 between pairs of wavelet filters with the same length. *(A, B)* Weighted network diagnostics including *(A)* mean correlation coefficient and *(B)* variance of correlation coefficients. *(C–F)* Binary network diagnostics calculated at a graph density of 25% obtained through a cumulative thresholding procedure, including *(C)* the clustering coefficient, *(D)* characteristic path length, *(E)* global efficiency, *(F)* local efficiency, *(G)* modularity index Q, and *(H)* the number of communities. Boxplots indicate the median and quartiles of the data acquired from 29 health subjects.

	Daubechies Extremal Phase (dF=9,252)		Daubechies Least Asymmetric (dF=6,168)		Coiflet (dF=3,84)	
	F	p	F	p	F	p
Mean correlation coefficient	27.97	0.0000	29.34	0.0000	32.31	0.0000
Variance of correlation coefficients	94.97	0.0000	102.80	0.0000	103.99	0.0000
Clustering coefficient	0.17	0.9966	0.50	0.8068	0.22	0.8820
Characteristic path length	0.15	0.9978	0.13	0.9923	0.46	0.7082
Global efficiency	8.60	0.0000	1.75	0.1129	4.52	0.0055
Local efficiency	2.01	0.0389	0.58	0.7451	1.77	0.1582
Modularity	0.39	0.9410	0.51	0.7976	0.11	0.9513
Number of communities	4.87	0.0000	2.18	0.0476	4.81	0.0039

Table 1. Effect of Wavelet Length. Results of Repeated Measures ANOVAs for network diagnostics extracted from 29 healthy controls at scale 1 and a graph density of 30%; network diagnostic is treated as a factor and wavelet length is treated as a repeated measure, separately for each wavelet fiter type. Effects that are significant at $p < 0.05$, uncorrected, are shown in red.

	Daubechies Extremal Phase (dF=9,252)		Daubechies Least Asymmetric (dF=6,168)		Coiflet (dF=3,84)	
	F	p	F	p	F	p
Mean correlation coefficient	0.58	0.8126	0.77	0.5963	0.87	0.4591
Variance of correlation coefficients	34.05	0.0000	52.72	0.0000	44.25	0.0000
Clustering coefficient	0.36	0.9512	0.37	0.8950	0.20	0.8995
Characteristic path length	0.67	0.7331	1.78	0.1061	0.73	0.5383
Global efficiency	0.82	0.5966	1.32	0.2522	1.06	0.3709
Local efficiency	0.67	0.7332	0.45	0.8424	0.23	0.8782
Modularity	0.21	0.9932	0.95	0.4588	0.24	0.8711
Number of communities	0.65	0.7555	1.21	0.3019	0.22	0.8806

Table 2. Effect of Wavelet Length. Results of Repeated Measures ANOVAs for network diagnostics extracted from 29 healthy controls at scale 3 and a graph density of 30%; network diagnostic is treated as a factor and wavelet length is treated as a repeated measure, separately for each wavelet fiter type. Effects that are significant at $p < 0.05$, uncorrected, are shown in red.

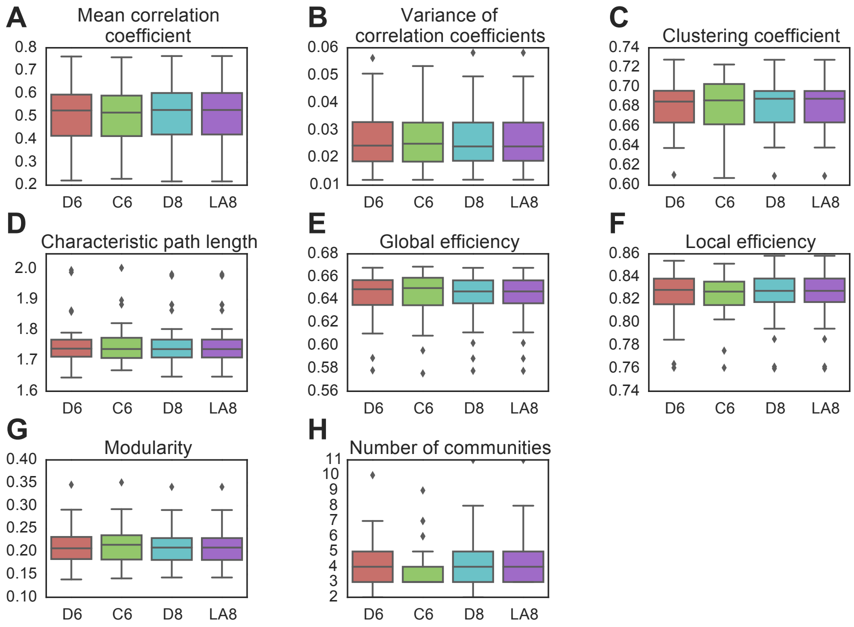

Fig 5. Effect of Wavelet Filter on Network Diagnostics in wavelet scale 2 between pairs of wavelet filters with the same length. *(A, B)* Weighted network diagnostics including *(A)* mean correlation coefficient and *(B)* variance of correlation coefficients. *(C–F)* Binary network diagnostics calculated at a graph density of 35% obtained through a cumulative thresholding procedure, including *(C)* the clustering coefficient, *(D)* characteristic path length, *(E)* global efficiency, *(F)* local efficiency, *(G)* modularity index Q, and *(H)* the number of communities. Boxplots indicate the median and quartiles of the data acquired from 29 health subjects.

Frequency Dependence of Assessed Group Differences

In the main manuscript, we present a set of analyses to assess group differences between healthy controls and schizophrenia patients in network diagnostic values obtained from functional brain networks constructed from scale 2 wavelet coefficients, corresponding approximately to the frequency range 0.06–0.12 Hz. We observed that group differences were most salient at longer wavelet lengths, across the 3 filter types, and especially for the following network diagnostics: mean correlation coefficient, variance of correlation coefficients, clustering coefficient, modularity, and number of communities.

Here, we ask whether these results are frequency dependent by assessing group differences in network diagnostic values obtained from functional brain networks constructed from scale 1 (approximately 0.125–0.25 Hz; see Fig. 6), scale 3 (approximately 0.03–0.06 Hz; see Fig. 7), and scale 4 (approximately 0.015–0.03 Hz; see Fig. 8) wavelet coefficients. Visually, we observe that group differences are more salient at smaller wavelet lengths than larger wavelet lengths in functional brain networks constructed from scale 1 coefficients, especially for the mean correlation coefficient, variance in correlation coefficients, local efficiency, and number of communities. Group differences are much weaker over all (for both long and short wavelets) in functional brain networks extracted from scale 3 and scale 4 coefficients, corresponding to BOLD signal variation in lower frequency bands (< 0.06 Hz).

Together, these results indicate that (i) group differences in functional brain network architecture can be differentially salient across frequency bands, and that (ii) the

optimal wavelet filter length may depend upon the frequency band of interest. In this dataset, we observe that longer wavelets are more sensitive to group differences in functional brain networks constructed from wavelet scale 2 coefficients, whose smoothness might facilitate a more sensitive characterization of low frequency fluctuations in the BOLD signal. In contrast, shorter wavelets are more sensitive to group differences in functional brain networks constructed from wavelet scale 1 coefficients, whose discrete nature might facilitate a more sensitive characterization of high frequency fluctuations in the BOLD signal.

Frequency Dependence of Classification Results

In the main text, we apply non-parametric machine learning techniques to determine whether different wavelet filters provide different degrees of classification accuracy. Specifically, we generated decision trees to classify healthy controls and people with schizophrenia based on graph metrics extracted from functional brain networks constructed from correlations in scale 2 wavelet coefficients. We observed that the classification accuracy ranged from approximately 63.8% to approximately 82.8%, the classification sensitivity ranged from approximately 65.5% to approximately 96.6%, and the classification specificity ranged from approximately 51.7% to 79.3%. Here we ask whether classification accuracy could be enhanced by including graph metric values from all four wavelet scales (scales 1–4). Interestingly, we observe that the classification accuracy tends to decrease with the addition of these features (see Table 4). Quantitatively, the average classification accuracy with scale 2 graph metric values was 73.80 with a standard deviation over wavelet lengths and types of 4.90, while the average classification accuracy with all four scales was 67.31 with a standard deviation of 4.70. Using a one-sample t-test, we found that the differences between the classification accuracies of scale 2 versus all four scales was significantly different from zero (one-sample t-test gives $t = 4.72$, $p = 1.30 \times 10^{-4}$), demonstrating that classification accuracy decreased with the addition of features from scales 1, 3, and 4. This decrease in classification accuracy could either be due to redundant information present across scales, or an increased difficulty in finding the optimal decision tree across many more variables.

Dependence of Assessed Group Differences on Methodological Choices

In the main manuscript, we observed that the p-values for parametric t-tests measuring differences in network diagnostic values between healthy controls and people with schizophrenia decreased with increasing wavelet length, suggesting that longer wavelets display greater statistical sensitivity to group differences in these data. Here in the SI, we explore the dependence of these results on methodological choices in network construction.

1. First, we examine the effect of the frequency band of interest. In the main manuscript, we assess functional brain networks constructed from scale 2 wavelet coefficients, and demonstrate that group differences in network diagnostics are most salient at longer wavelet lengths. Here we show that group differences in network diagnostics extracted from functional brain networks constructed from scale 1 wavelet coefficients are more salient at short wavelet lengths (see Fig. 9). Therefore, group differences are differentially assessed across frequency bands.

2. Second, we examine the effect of the measure of functional connectivity, including partial correlation (Fig. 10 and 11), wavelet coherence (see Supplemental Methods;

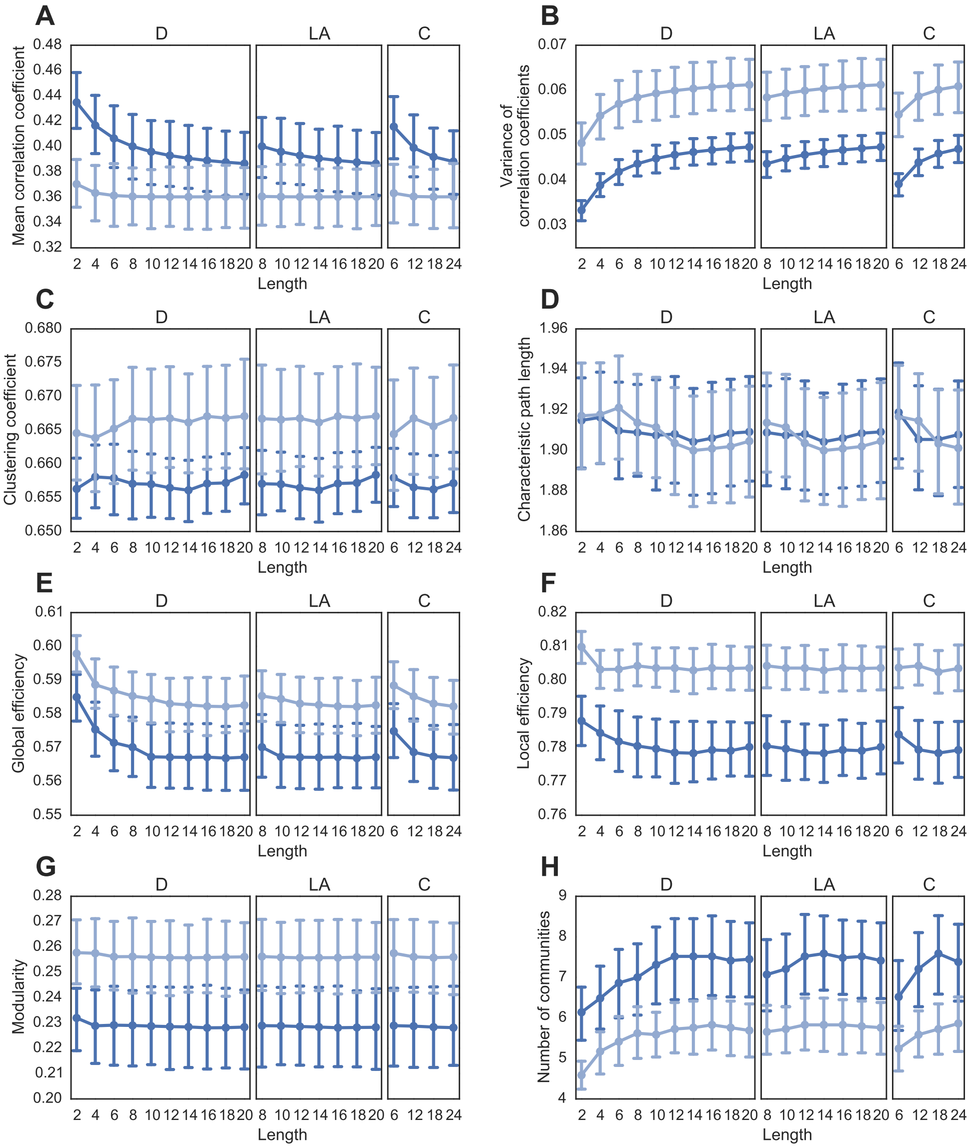

Fig 6. Effect of Wavelet Length on Network Diagnostics in wavelet scale 1 for all wavelet filters. *(A, B)* Weighted network diagnostics including *(A)* mean correlation coefficient and *(B)* variance of correlation coefficients. *(C–F)* Binary network diagnostics calculated at a graph density of 30% obtained through a cumulative thresholding procedure, including *(C)* the clustering coefficient, (*(D)* characteristic path length, *(E)* global efficiency, *(F)* local efficiency, *(G)* modularity index Q, and *(H)* the number of communities. The more saturated curves represent data from the 29 healthy controls, while the less saturated curves represent data from 29 people with schizophrenia. Error bars depict standard errors of the mean across subjects.

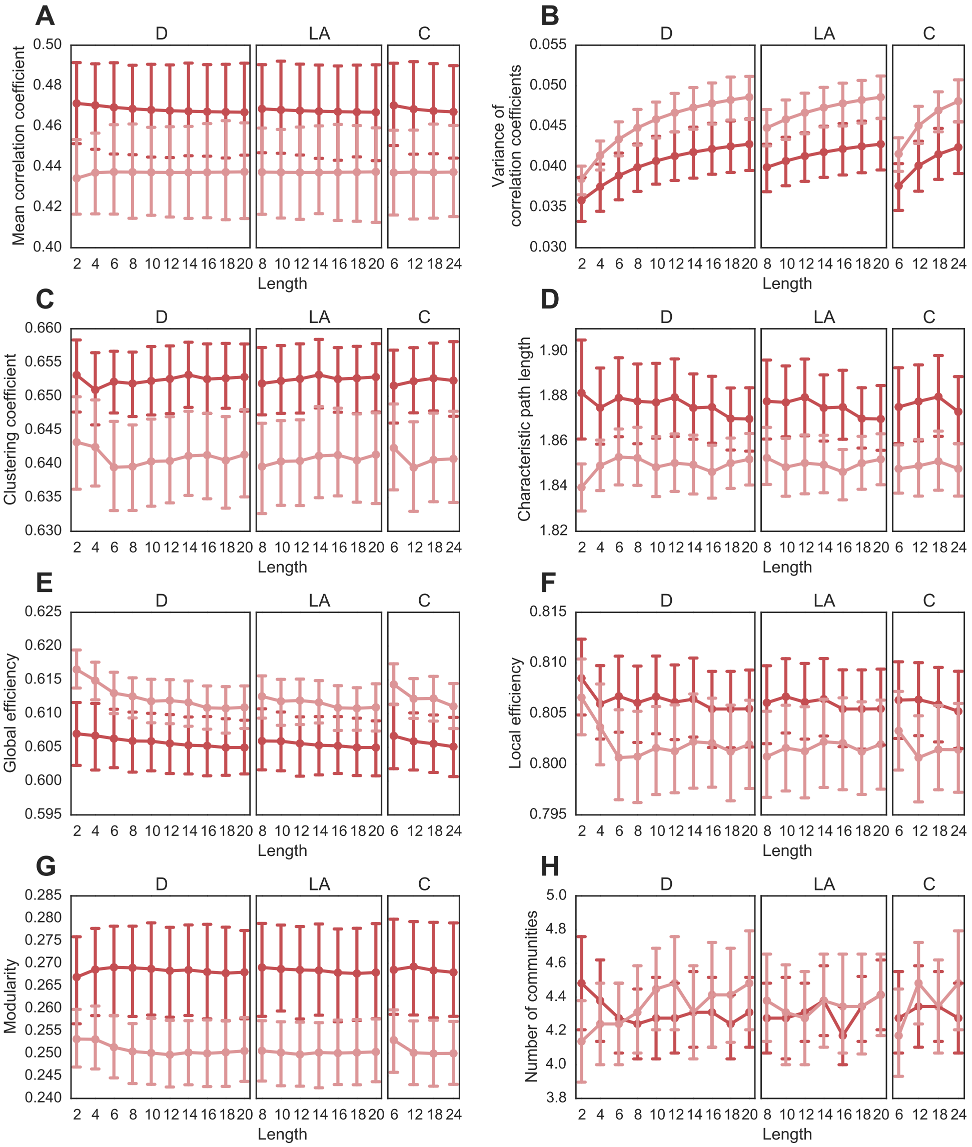

Fig 7. Effect of Wavelet Length on Network Diagnostics in wavelet scale 3 for all wavelet filters. *(A, B)* Weighted network diagnostics including *(A)* mean correlation coefficient and *(B)* variance of correlation coefficients. *(C–F)* Binary network diagnostics calculated at a graph density of 30% obtained through a cumulative thresholding procedure, including *(C)* the clustering coefficient, (*(D)* characteristic path length, *(E)* global efficiency, *(F)* local efficiency, *(G)* modularity index Q, and *(H)* the number of communities. The more saturated curves represent data from the 29 healthy controls, while the less saturated curves represent data from 29 people with schizophrenia. Error bars depict standard errors of the mean across subjects.

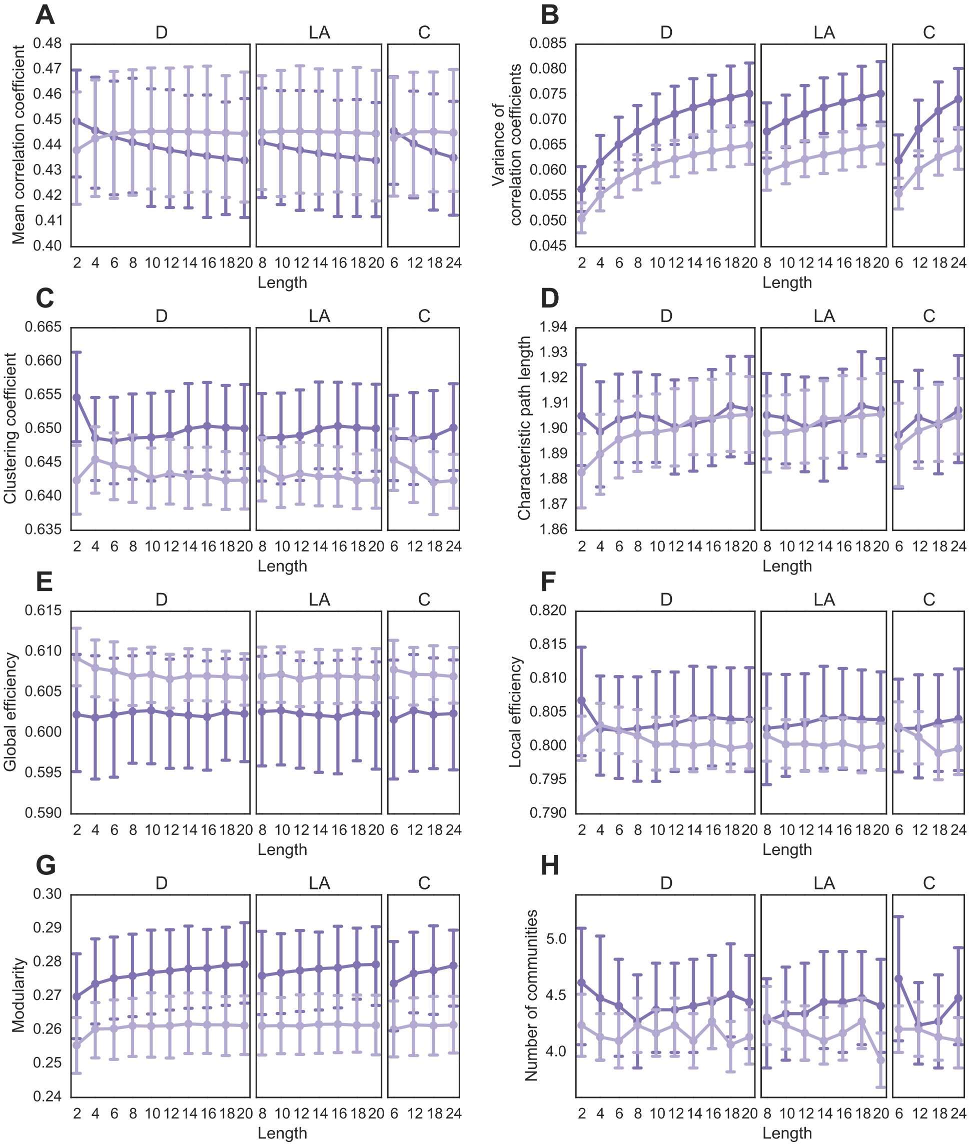

Fig 8. Effect of Wavelet Length on Network Diagnostics in wavelet scale 4 for all wavelet filters. *(A, B)* Weighted network diagnostics including *(A)* mean correlation coefficient and *(B)* variance of correlation coefficients. *(C–F)* Binary network diagnostics calculated at a graph density of 30% obtained through a cumulative thresholding procedure, including *(C)* the clustering coefficient, (*(D)* characteristic path length, *(E)* global efficiency, *(F)* local efficiency, *(G)* modularity index Q, and *(H)* the number of communities. The more saturated curves represent data from the 29 healthy controls, while the less saturated curves represent data from 29 people with schizophrenia. Error bars depict standard errors of the mean across subjects.

Fig. 12 and 13), and wavelet correlation (see main manuscript). We observe that the effect of wavelet length is more salient when using wavelet correlation than when using wavelet coherence or partial correlation.

3. Third, we examine the effect of the strength of edges. In the main manuscript, we examined networks constructed from the 30% strongest connections. Here, we ask whether networks constructed from weak connections might provide complementary information, as recently proposed in the literature [?, ?]. Specifically, we examine group differences in network diagnostics extracted from functional brain networks constructed from the 30% weakest (see Fig. 14 and 15), 10% weakest (Fig. 16 and 17), and 1% weakest (Fig. 18 and 19) connections, where connectivity is defined as the wavelet correlation between scale 1 or 2 wavelet coefficients. We observe that the effect of wavelet length is more salient when using the strongest 30% connections or 10% weakest connections than when using the 30% or 1% weakest connections.

4. Fourth, we examine the effect of choosing wavelet details (see Supplemental Methods) over wavelet coefficients (see Fig. 20 and 21). Results are consistent across the use of both wavelet details and wavelet coefficients.

In summary, we observe that the impact of wavelet length on group differences is (i) dependent on frequency, (ii) more salient when using wavelet correlation than when using wavelet coherence or partial correlation, (iii) more salient when using the strongest 30% connections or 10% weakest connections than when using the 30% or 1% weakest connections, and (iv) relatively agnostic to the use of either wavelet details or wavelet coefficients.

	Daubechies Extremal Phase (dF=9,252)		Daubechies Least Asymmetric (dF=6,168)		Coiflet (dF=3,84)	
	F	p	F	p	F	p
Mean correlation coefficient	4.81	0.0000	7.52	0.0000	5.65	0.0014
Variance of correlation coefficients	92.21	0.0000	106.66	0.0000	105.99	0.0000
Clustering coefficient	1.64	0.1052	0.84	0.5396	0.33	0.8037
Characteristic path length	0.35	0.9571	0.58	0.7495	0.65	0.5839
Global efficiency	0.19	0.9948	1.12	0.3514	0.58	0.6276
Local efficiency	1.01	0.4310	0.54	0.7781	0.29	0.8302
Modularity	5.30	0.0000	5.76	0.0000	4.27	0.0074
Number of communities	0.86	0.5587	1.11	0.3607	2.20	0.0941

Table 3. Effect of Wavelet Length. Results of Repeated Measures ANOVAs for network diagnostics extracted from 29 healthy controls at scale 4 and a graph density of 30%; network diagnostic is treated as a factor and wavelet length is treated as a repeated measure, separately for each wavelet fiter type. Effects that are significant at $p < 0.05$, uncorrected, are shown in red.

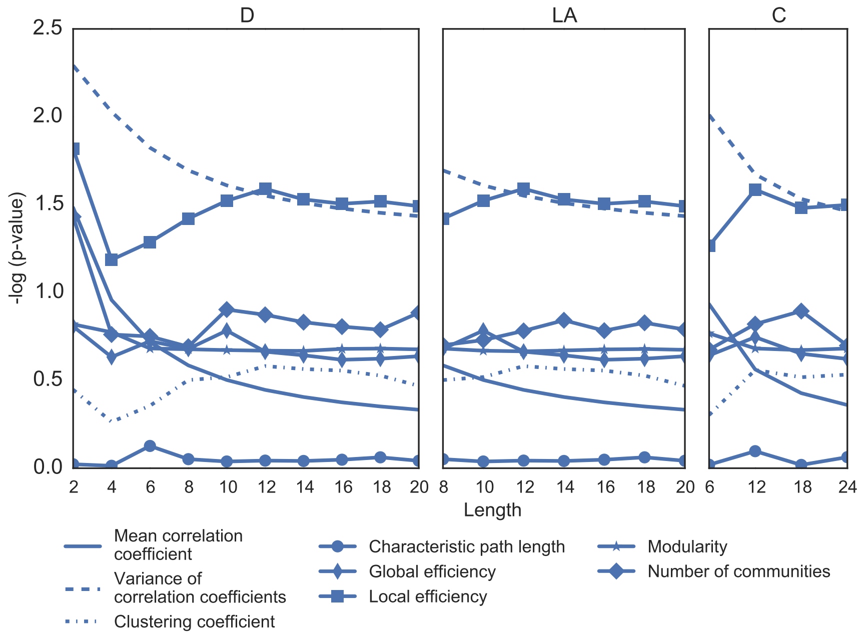

Fig 9. Effect of Wavelet Filter Type and Length on Statistical Sensitivity in Group Comparisons. Negative common logarithm of the p-values obtained from two-sample t-tests between diagnostic values extracted from healthy control networks versus those extracted from schizophrenia patient networks. Network diagnostics are calculated for functional brain networks constructed from scale 1 wavelet coefficients. Networks represent the strongest 30% of edges.

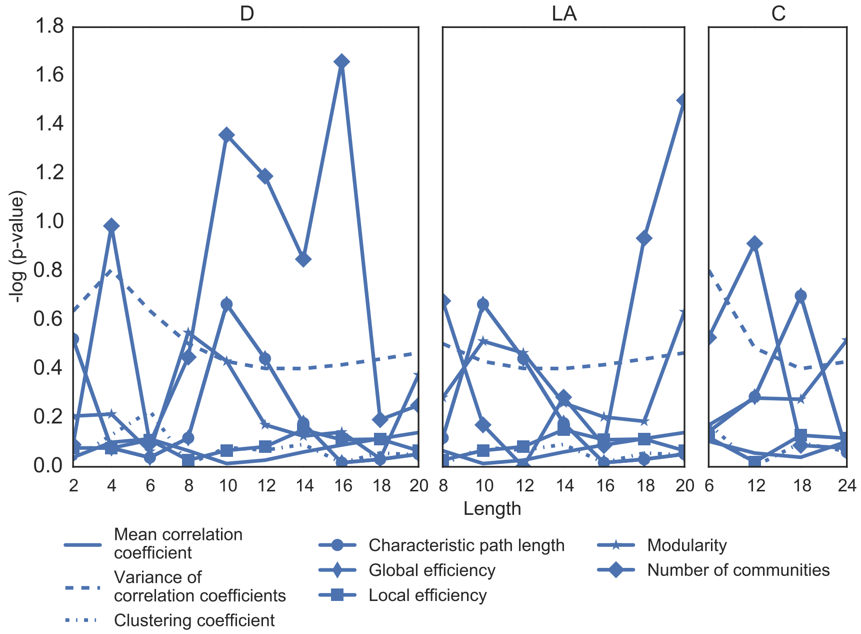

Fig 10. Effect of Wavelet Filter Type and Length on Statistical Sensitivity in Group Comparisons. Negative common logarithm of the p-values obtained from two-sample t-tests between diagnostic values extracted from healthy control networks versus those extracted from schizophrenia patient networks. Network diagnostics are calculated for functional brain networks constructed from scale 1 wavelet coefficients. Networks represent the strongest 30% of edges, and functional connectivity is calculated from partial correlations in wavelet coefficients.

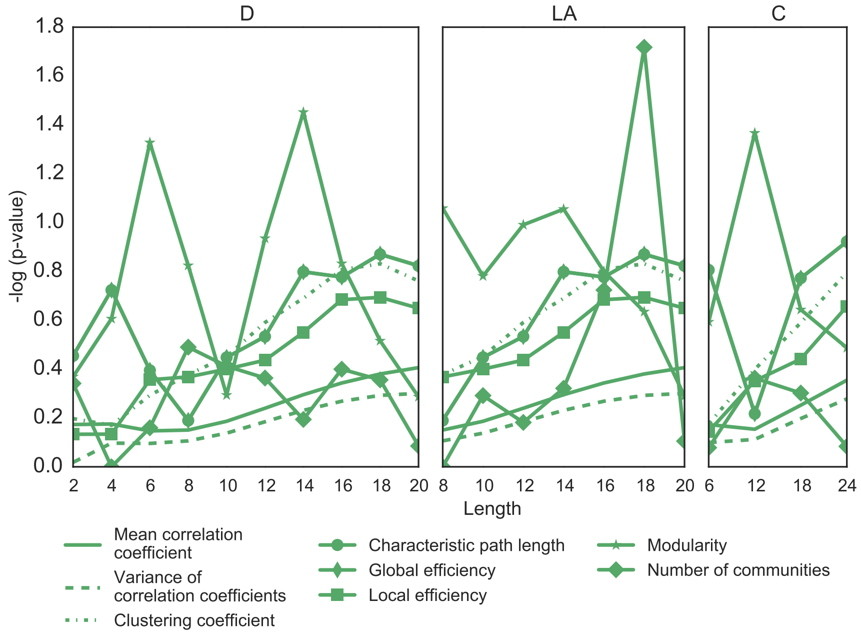

Fig 11. Effect of Wavelet Filter Type and Length on Statistical Sensitivity in Group Comparisons. Negative common logarithm of the p-values obtained from two-sample t-tests between diagnostic values extracted from healthy control networks versus those extracted from schizophrenia patient networks. Network diagnostics are calculated for functional brain networks constructed from scale 2 wavelet coefficients. Networks represent the strongest 30% of edges, and functional connectivity is calculated from partial correlations in wavelet coefficients.

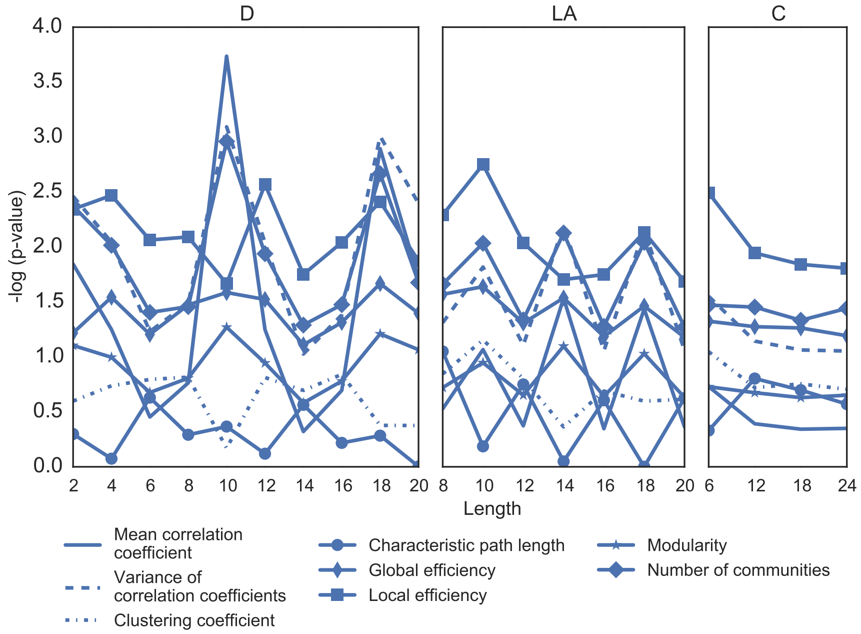

Fig 12. Effect of Wavelet Filter Type and Length on Statistical Sensitivity in Group Comparisons. Negative common logarithm of the p-values obtained from two-sample t-tests between diagnostic values extracted from healthy control networks versus those extracted from schizophrenia patient networks. Network diagnostics are calculated for functional brain networks constructed from scale 1 wavelet coefficients. Networks represent the strongest 30% of edges, and functional connectivity is calculated from wavelet coherence.

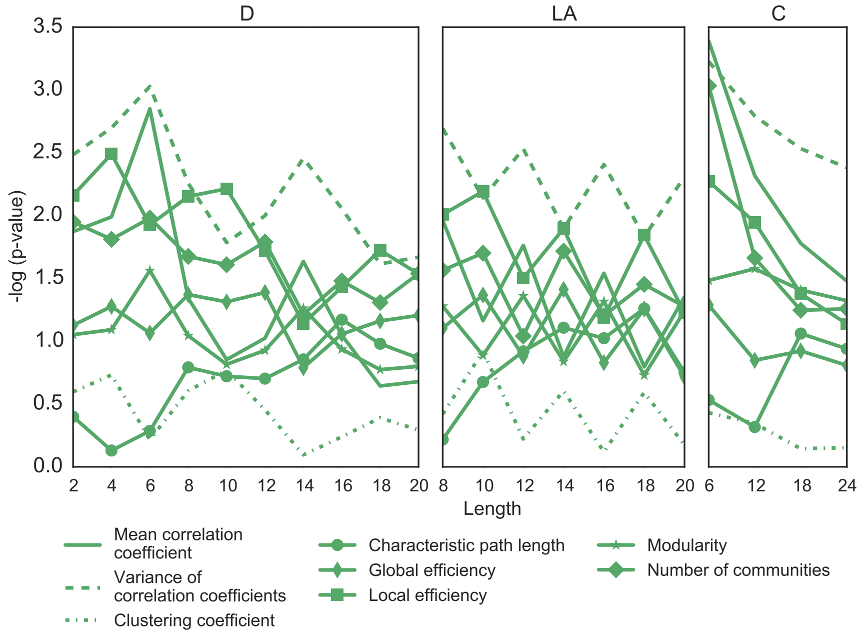

Fig 13. Effect of Wavelet Filter Type and Length on Statistical Sensitivity in Group Comparisons. Negative common logarithm of the p-values obtained from two-sample t-tests between diagnostic values extracted from healthy control networks versus those extracted from schizophrenia patient networks. Network diagnostics are calculated for functional brain networks constructed from scale 2 wavelet coefficients. Networks represent the strongest 30% of edges, and functional connectivity is calculated from wavelet coherence.

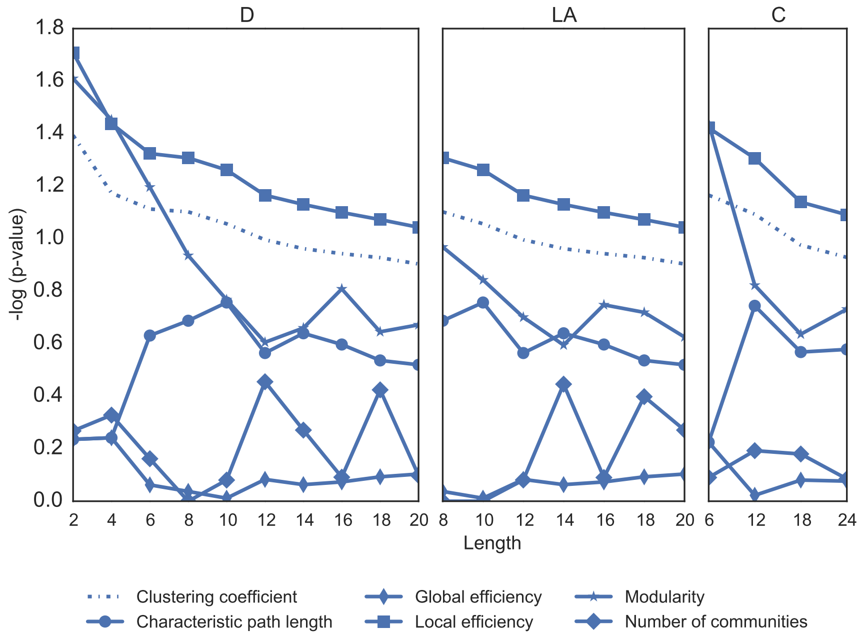

Fig 14. Effect of Wavelet Filter Type and Length on Statistical Sensitivity in Group Comparisons. Negative common logarithm of the p-values obtained from two-sample t-tests between diagnostic values extracted from healthy control networks versus those extracted from schizophrenia patient networks. Network diagnostics are calculated for functional brain networks constructed from scale 1 wavelet coefficients. Networks represent the weakest 30% of edges.

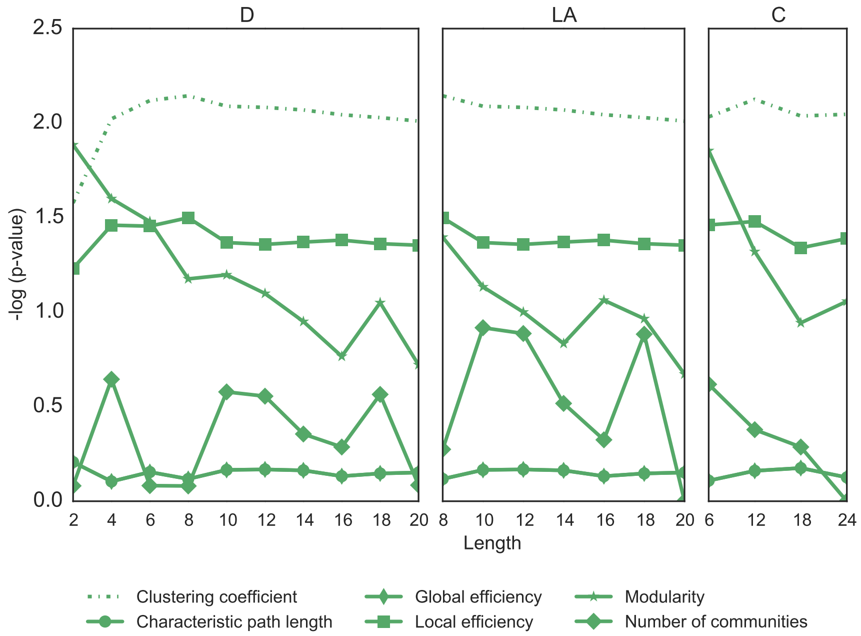

Fig 15. Effect of Wavelet Filter Type and Length on Statistical Sensitivity in Group Comparisons. Negative common logarithm of the p-values obtained from two-sample t-tests between diagnostic values extracted from healthy control networks versus those extracted from schizophrenia patient networks. Network diagnostics are calculated for functional brain networks constructed from scale 2 wavelet coefficients. Networks represent the weakest 30% of edges.

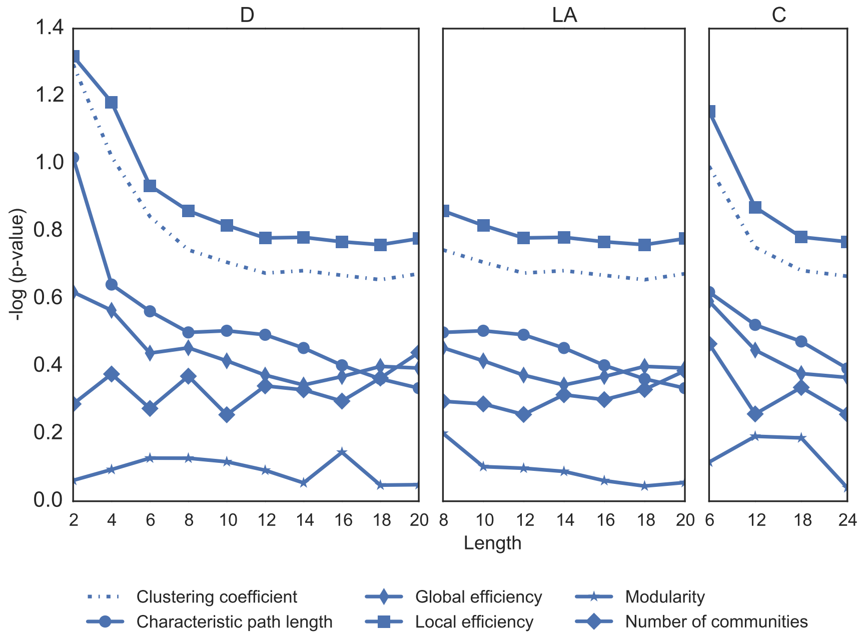

Fig 16. Effect of Wavelet Filter Type and Length on Statistical Sensitivity in Group Comparisons. Negative common logarithm of the p-values obtained from two-sample t-tests between diagnostic values extracted from healthy control networks versus those extracted from schizophrenia patient networks. Network diagnostics are calculated for functional brain networks constructed from scale 1 wavelet coefficients. Networks represent the weakest 10% of edges.

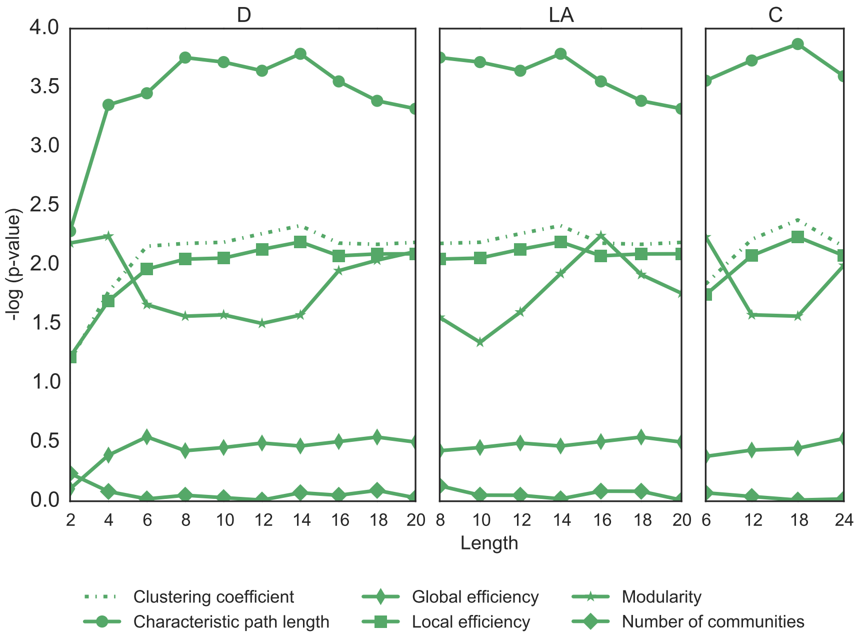

Fig 17. Effect of Wavelet Filter Type and Length on Statistical Sensitivity in Group Comparisons. Negative common logarithm of the p-values obtained from two-sample t-tests between diagnostic values extracted from healthy control networks versus those extracted from schizophrenia patient networks. Network diagnostics are calculated for functional brain networks constructed from scale 2 wavelet coefficients. Networks represent the weakest 10% of edges.

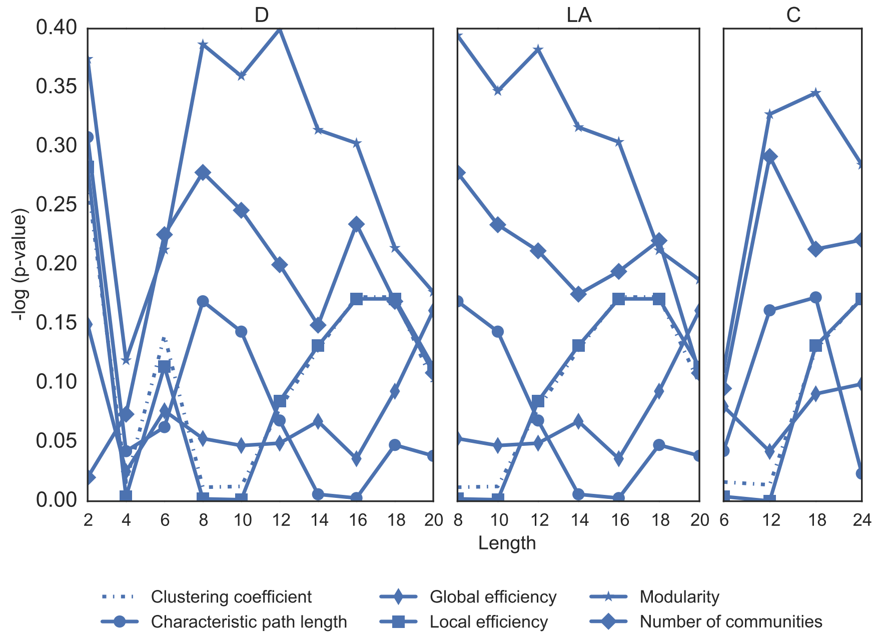

Fig 18. Effect of Wavelet Filter Type and Length on Statistical Sensitivity in Group Comparisons. Negative common logarithm of the p-values obtained from two-sample t-tests between diagnostic values extracted from healthy control networks versus those extracted from schizophrenia patient networks. Network diagnostics are calculated for functional brain networks constructed from scale 1 wavelet coefficients. Networks represent the weakest 1% of edges..

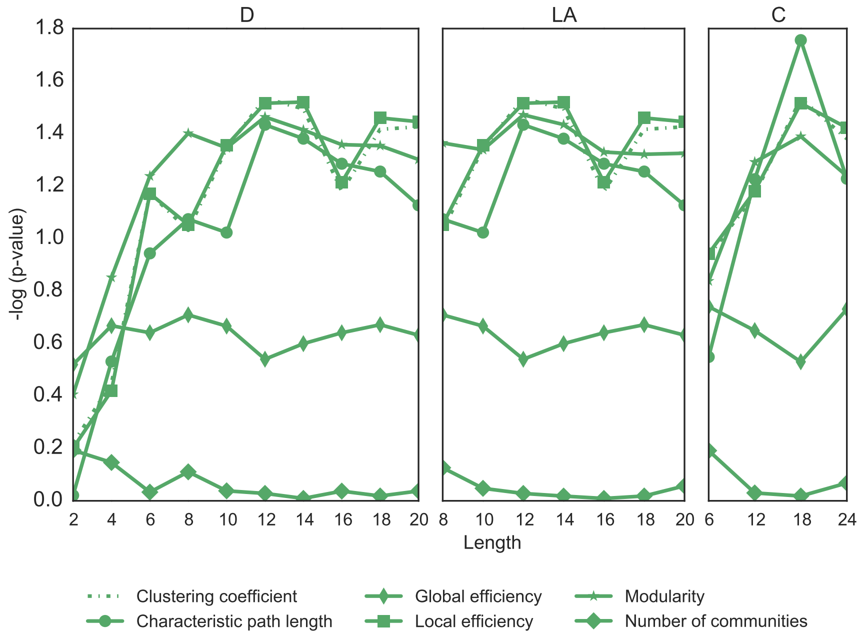

Fig 19. Effect of Wavelet Filter Type and Length on Statistical Sensitivity in Group Comparisons. Negative common logarithm of the p-values obtained from two-sample t-tests between diagnostic values extracted from healthy control networks versus those extracted from schizophrenia patient networks. Network diagnostics are calculated for functional brain networks constructed from scale 2 wavelet coefficients. Networks represent the weakest 1% of edges.

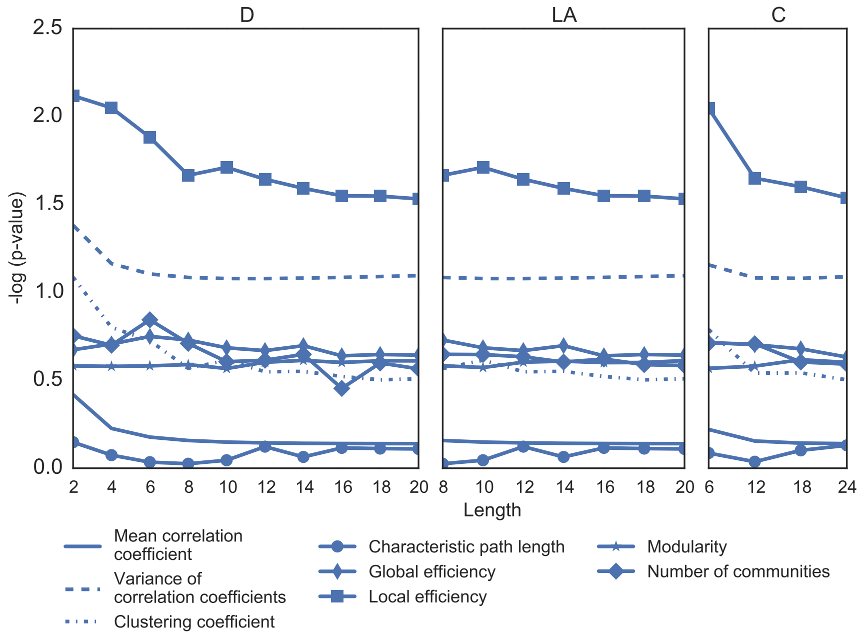

Fig 20. Effect of Wavelet Filter Type and Length on Statistical Sensitivity in Group Comparisons. Negative common logarithm of the p-values obtained from two-sample t-tests between diagnostic values extracted from healthy control networks versus those extracted from schizophrenia patient networks. Network diagnostics are calculated for functional brain networks constructed from scale 1 **detail** coefficients. Networks represent the strongest 30% of edges.

D. Extremal Phase									
D2	D4	D6	D8	D10	D12	D14	D16	D18	D20
63.8	67.2	65.5	74.1	74.1	72.4	77.6	69.0	79.3	72.4
66.9	56.9	68.9	65.7	70.4	61.9	65.7	70.7	74.3	67.4
D. Least Asymmetric									
LA8	LA10	LA12	LA14	LA16	LA18	LA20			
74.1	79.3	75.9	82.8	77.6	79.3	70.7			
72.8	68.7	63.7	58.3	67.4	72.0	64.1			
Coiflet									
C6	C12	C18	C24						
72.4	70.7	77.6	74.1						
72.2	63.5	72.0	70.0						

Table 4. Classification accuracy when using graph metric values from scale 2 (upper rows of each set) and from scales 1 through 4 (lower rows of each set). Results are separate by wavelet family: D (Daubechies Extremal Phase or D. Extremal Phase), LA (Daubechies Least Asymmetric or D. Least Asymmetric), and C (Coiflet).

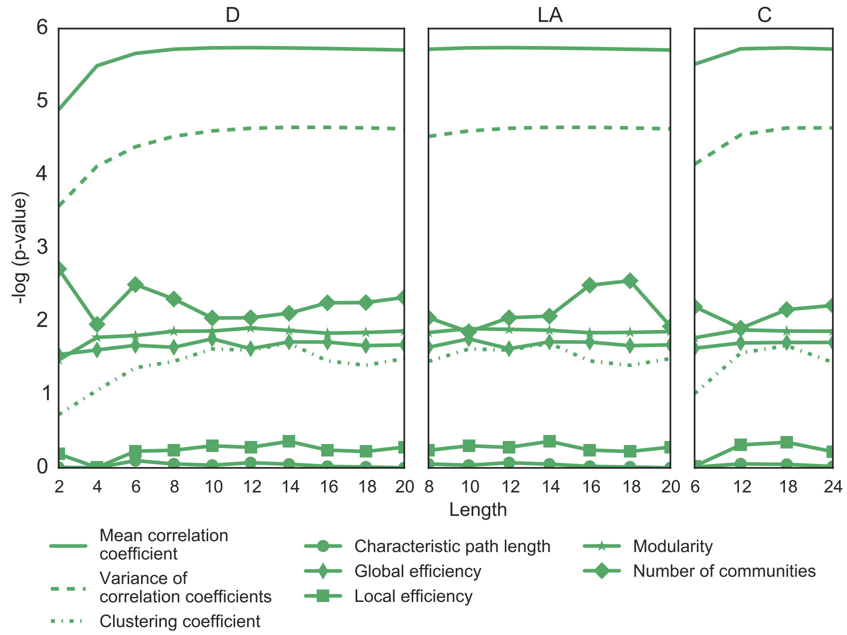

Fig 21. Effect of Wavelet Filter Type and Length on Statistical Sensitivity in Group Comparisons. Negative common logarithm of the p-values obtained from two-sample t-tests between diagnostic values extracted from healthy control networks versus those extracted from schizophrenia patient networks. Network diagnostics are calculated for functional brain networks constructed from scale 2 **detail** coefficients. Networks represent the strongest 30% of edges.
